# Chronic Wasting Disease: State of the Science

**DOI:** 10.3390/pathogens13020138

**Published:** 2024-02-02

**Authors:** Jason C. Bartz, Rebeca Benavente, Byron Caughey, Sonja Christensen, Allen Herbst, Edward A. Hoover, Candace K. Mathiason, Debbie McKenzie, Rodrigo Morales, Marc D. Schwabenlander, Daniel P. Walsh, the NC1209: North American Interdisciplinary Chronic Wasting Disease Research Consortium Members

**Affiliations:** 1 Department of Medical Microbiology and Immunology, Creighton University, Omaha, NE 68178, USA; jbartz@creighton.edu; 2 Department of Neurology, The University of Texas Health Science Center at Houston, Houston, TX 77030, USA; rebecabenaventegh@gmail.com (R.B.); rodrigo.moralesloyola@uth.tmc.edu (R.M.); 3 Laboratory of Neurological Infections and Immunity, Rocky Mountain Laboratories, National Institute of Allergy and Infectious Diseases, National Institutes of Health, Hamilton, MT 59840, USA; bcaughey@nih.gov; 4 Department of Fisheries and Wildlife, Michigan State University, East Lansing, MI 48824, USA; chris625@msu.edu; 5 U.S. Geological Survey, National Wildlife Health Center, Madison, WI 53711, USA; aherbst@usgs.gov; 6 Prion Research Center, Department of Microbiology, Immunology and Pathology, Colorado State University, Fort Collins, CO 80523, USA; edward.hoover@colostate.edu (E.A.H.); candace.mathiason@colostate.edu (C.K.M.); 7 Department of Biological Sciences, Centre for Prions and Protein Folding Diseases, University of Alberta, Edmonton, AB T6G 2M9, Canada; debbie.mckenzie@ualberta.ca; 8 Centro Integrativo de Biologia y Quimica Aplicada (CIBQA), Universidad Bernardo O’Higgins, Santiago 8370993, Chile; 9 Minnesota Center for Prion Research and Outreach, Department of Veterinary and Biomedical Sciences, College of Veterinary Medicine, University of Minnesota, St. Paul, MN 55108, USA; schwa239@umn.edu; 10 U.S. Geological Survey, Montana Cooperative Wildlife Research Unit, University of Montana, Missoula, MT 59812, USA

**Keywords:** prion disease, cervids, RT-QuIC, PMCA, interspecies transmission, zoonosis, environmental prions

## Abstract

Chronic wasting disease (CWD) is a prion disease affecting cervid species, both free-ranging and captive populations. As the geographic range continues to expand and disease prevalence continues to increase, CWD will have an impact on cervid populations, local economies, and ecosystem health. Mitigation of this “wicked” disease will require input from many different stakeholders including hunters, landowners, research biologists, wildlife managers, and others, working together. The NC1209 (North American interdisciplinary chronic wasting disease research consortium) is composed of scientists from different disciplines involved with investigating and managing CWD. Leveraging this broad breadth of expertise, the Consortium has created a state-of-the-science review of five key aspects of CWD, including current diagnostic capabilities for detecting prions, requirements for validating these diagnostics, the role of environmental transmission in CWD dynamics, and potential zoonotic risks associated with CWD. The goal of this review is to increase stakeholders’, managers’, and decision-makers’ understanding of this disease informed by current scientific knowledge.

## 1. Introduction

Chronic wasting disease (CWD), an emerging disease of cervids (e.g., deer, elk, and moose), was first identified in the late 1960s [1] in Colorado and Wyoming and has subsequently been identified in captive and free-ranging cervids in 32 U.S. States, 4 Canadian provinces, the Republic of South Korea, and Scandinavia. The geographic distribution of CWD continues to expand, and the prevalence of CWD within certain free-ranging cervid populations approaches 85% (https://www.saskatchewan.ca/residents/environment-public-health-and-safety/wildlife-issues/fish-and-wildlife-diseases/chronic-wasting-disease/cwd-map, accessed on 15 January 2024). Prions persist in the environment for years, facilitating indirect transmission between cervids and further complicating mitigation strategies. Overall, these combined factors have resulted in CWD having detrimental effects on cervid populations and associated industries.

CWD belongs to a group of diseases that are caused by infectious proteins called prions, which lack a specific nucleic acid genome, unlike viruses and other pathogens [2,3]. Prion replication occurs when the infectious form of the prion protein, PrP^Sc^, binds to and converts the normal form of the host-encoded prion protein, PrP^C^, to PrP^Sc^ [4,5,6,7]. Compared to conventional pathogens such as viruses, relatively little is understood about the details of how prions replicate, spread between animals, and cause disease. However, they infect a wide range of species including humans, are inevitably fatal, and have no known therapies or cures. It is also known that prions from one species can infect new species (e.g., transmission of bovine spongiform encephalopathy (BSE) or “mad cow” disease to humans); yet, the species host range of CWD is unknown. There are 11.4 million deer hunters in the USA (https://www.deeranddeerhunting.com/content/blogs/dan-schmidt-deer-blog-whitetail-wisdom/how-many-deer-hunters-in-the-united-states, accessed on 30 January 2024), and it has been estimated that up to 15,000 CWD-infected cervids are consumed annually (https://mwf.mb.ca/wp-content/uploads/2021/11/CWD-Comprehensive-Analysis-Supplement-PR.pdf, accessed on 30 January 2024). These data underestimate the expanding rates of exposure. For example, in 2022, 1420 white-tailed deer tested positive for CWD in the southern farmland zone of Wisconsin, and the prevalence was 18.4%. The 2022 harvest in the southern farmland zone was 72,933 deer and if the sampling was unbiased, it would indicate the exposure of at least 13,420 individuals to CWD infectivity (https://apps.dnr.wi.gov/cwd/summary/zone, accessed on 15 January 2024). Many hunters do not submit deer for testing, even when the deer is harvested from an area with a high prevalence of CWD.

Additionally, the geographic range of CWD overlaps with other species that may be susceptible to CWD via contact with cervids or CWD-contaminated environments (e.g., feral swine) [8]. If left unabated, CWD poses a threat to cervid populations, poses a risk to human and animal health, and threatens long-standing hunting and farming traditions and the economic benefits that come with them to both private citizens and government agencies.

Combating this bourgeoning disease requires the cooperation of individuals and groups with wide-ranging scientific and management expertise from across many jurisdictions. The NC1209: North American Interdisciplinary Chronic Wasting Disease Research Consortium (herein, the consortium) was established as a Multistate Research Project with support from the United States Department of Agriculture (USDA) to advance the scientific understanding of the etiology and management of CWD through collaborative research. The consortium currently focuses its efforts on the development of novel CWD diagnostic, treatment, and mitigation strategies; effective public communication; and coordination of management strategies across agencies.

The long-term goal of the consortium is the reduction of CWD in North America. The benefits of the consortium are the communication, development of ideas, and collaboration between the members which would not have been possible without this guided cooperative. These efforts build a foundation for science-driven advances in the fight against CWD in North America and throughout the world. This interdisciplinary group of CWD experts leverages their combined knowledge as a scientific resource for wildlife management agencies, policymakers, and the public. As part of the consortium’s mandate, members have compiled a series of brief overviews of existing knowledge of current topics in CWD research, including (i) providing information on current methods in CWD detection (protein misfolding cyclic amplification and real-time quaking-induced conversion), (ii) the path to using these in vitro assays as an officially approved USDA diagnostic test, (iii) the current knowledge on CWD in the environment, and (iv) the potential for zoonotic transmission.

## 2. Protein Misfolding Cyclic Amplification

Compelling evidence from human and animal prionopathies indicates that the best biomarker for these diseases is the infectious form of the prion protein (PrP^Sc^) [9,10]. Hence, the CWD status of an animal is currently evaluated post-mortem in brain and/or lymph nodes where infectious prions accumulate in relatively high concentrations (https://www.aphis.usda.gov/animal_health/animal_diseases/cwd/downloads/cwd-program-standards.pdf, accessed on 30 January 2024). PrP^Sc^ is also present in quantities too low to be detected by conventional immunodetection methods in more easily accessible biological samples. Consequently, one of the main concerns involving CWD is the lack of sensitive pre-mortem diagnostic methods. To address this major limitation, techniques that either increase the sensitivity or the concentration of PrP^Sc^ have been developed. Two useful methodologies have been extensively developed and used by prion scientists, namely the protein misfolding cyclic amplification (PMCA) [11] and real-time quaking-induced conversion (RT-QuIC) [12].

**Figure 1:**
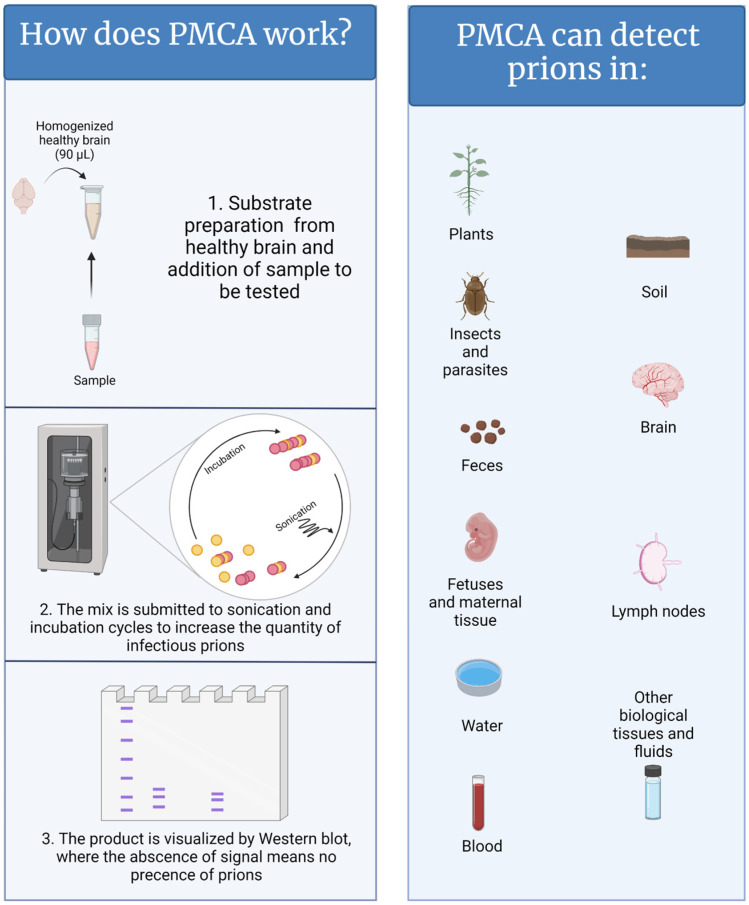
A graphical representation of the PMCA methods and samples where CWD has been detected. Figure generated by BioRender.

In contrast to natural and/or experimental animal infections, which can take months to decades, PMCA replicates disease-associated prions in just a few hours [13]. In a PMCA reaction, samples suspected to contain PrP^Sc^ are mixed with brain extracts from naïve animals (usually transgenic mice that have been genetically modified to express the healthy prion protein of the desired animal species) as a source of normally folded prion protein (PrP^C^) (Figure 1) [11,14]. In other words, PrP^C^ acts as the substrate for the generation of new PrP^Sc^. To accelerate the production of disease-associated prions, the PMCA mixture is subjected to cycles of incubation and sonication that facilitate protein misfolding, generating new active PrP^Sc^ “seeds”. When the PMCA process is concluded, the PMCA-generated PrP^Sc^ molecules are abundant and can be easily visualized by conventional techniques such as western blot [11]. PMCA reactions supplemented with samples lacking CWD prions fail to amplify PrP^Sc^. PMCA is highly efficient; it is estimated that a single aggregate or “seed” of infectious prions, orders of magnitude below the level needed to infect an animal, can be successfully detected by this technique [15,16,17]. In that respect, research and diagnostic laboratories are assessing the power of PMCA to diagnose CWD [18,19,20,21,22,23,24,25]. Published data demonstrate that PMCA is an excellent diagnostic method for detecting prions in samples derived from CWD-infected animals at pre-symptomatic stages of disease [10]. Some of the tested specimens, such as blood, saliva, feces, urine, muscle, and rectal mucosa are good candidates for the diagnosis of live animals. In parallel, the sensitivity and specificity of this technique in several sample types are being compared with conventional enzyme-linked immunosorbent assay (ELISA)-based methods and RT-QuIC. Importantly, the uses of PMCA go beyond animal-derived samples, including environmental components such as mineral licks, soils, plants, and human-made materials [22,23,26,27,28] and, therefore, may facilitate the diagnosis of CWD in animal populations and the environments that contain them.

Beyond diagnosis, PMCA also has great utility for elucidating disease mechanisms. PMCA has been used to demonstrate the presence of infectious prions in the semen and fetuses of CWD-infected animals [20,21,29], supporting (but not confirming) the idea that CWD can be transmitted by insemination and from mother to offspring [30]. This method has been used to model potential inter-species transmission of CWD [31]. Moreover, amplification of PrP^Sc^ via PMCA usually retains the strain properties of the infectious input PrP^Sc^ [32,33], facilitating the rapid detection of potentially different CWD strains. This is relevant as different prion strains manifest with varying rates of infection, altered prevalence in contaminated environments, and diverse potentials for inter-species infections [34,35].

As mentioned above, RT-QuIC is another prion replication method that has been extensively explored for its use as a CWD diagnostic platform. When compared with RT-QuIC, PMCA has advantages and disadvantages. Disadvantages of PMCA include: (i) PMCA’s more complex nature requires experienced technicians; (ii) the reaction times, from start to completion, are longer than RT-QuIC; (iii) costs are higher, as some PMCA components are more expensive than those used in RT-QuIC; (iv) the ethical issues of using brains from rodents as the key reagent in PMCA compared to proteins collected from bacteria in RT-QuIC; and (v) PMCA generates additional infectious prions that pose laboratory contamination. However, PMCA also displays advantages over RT-QuIC, namely: (i) its ability to more often discriminate among different strains of the infectious agent; (ii) its ability to generate infectious prions, which is useful for subsequent characterizations; (iii) its sensitivity to species-specific PRNP sequences (including polymorphic variations); and (iv) the ability of PMCA to detect prions in samples difficult to work with in RT-QuIC (e.g., blood).

Overall, PMCA and RT-QuIC are complementary. Their future development is expected to greatly help in understanding CWD. Moreover, PMCA looks promising for properly diagnosing CWD as well as to evaluate its dynamics in the environment.

## 3. Real-Time Quaking-Induced Conversion (RT-QuIC)

**Figure 2:**
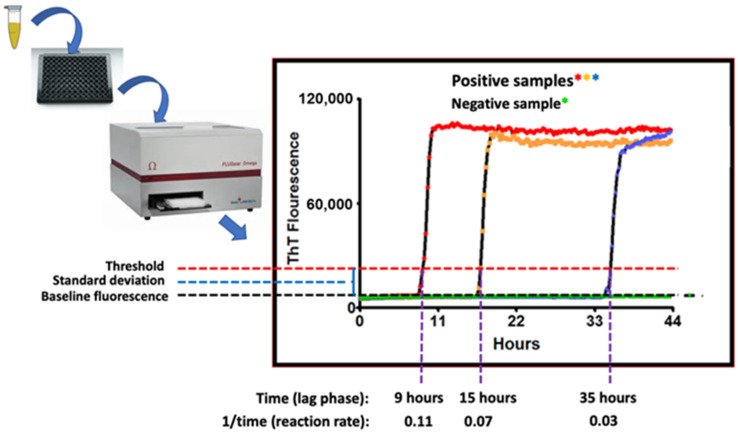
Schematic of the RT-QuIC assay readout. Annotated are time-dependent threshold crossings, or lack thereof, with the fluorescent readings taken at each time point illustrated by the colored asterisks where red, orange and blue asterisks represent readings of positive samples and green asterisks represent reading of the negative sample.

Real-time quaking-induced conversion (RT-QuIC) is a seeded amplification assay developed in the Atarashi and Caughey laboratories [36,37]. RT-QuIC is used to sensitively detect CWD (and other) prions in tissues, body fluids, and excretions. Prions (PrP^Sc^) coerce the misfolding of normal PrP^C^ proteins to the PrP^Sc^ conformation. This process ignites a misfolding chain reaction that spreads within cells, to other cells, to the blood, and to cells in distant organs. As PrP^Sc^ molecules are generated, they polymerize to form microfibrils with an amyloid structure [4]. RT-QuIC detects the growing PrP^Sc^ amyloid fibrils by use of the amyloid-binding benzothiazole dye, thioflavin T (ThT) [36,37]. RT-QuIC angles for prion seeds using a recombinant normal prion protein (PrP^C^) as the substrate/bait. The assay is performed in replicate microtiter plate wells (from 8 to 12 wells/sample) that are incubated, intermittently shaken, and analyzed in a fluorimeter/shaker/incubator (Figure 2). The growth of the prion-seeded PrP amyloid fibrils are detected in real-time by the binding of ThT, the fluorescence emission of which shifts to 480 nm.

Positive RT-QuIC reactions are recognized when the 480-nm fluorescence emission signal crosses a control threshold, typically the negative control level plus 5–10 standard deviations [36,37]. In general, the time to threshold is inversely proportional to the concentration of prion seeding activity in the test sample [36,38]. This relationship, or end-point dilutions [36], can be used to obtain quantitative comparisons of prion seeding activity between samples. For most diagnostic tissue samples (e.g., brain, lymph node), positive results can often be read within 12 h. For lower prion seed concentration samples, however, the inflection point can be as long as 48 h. Sample readout as positive, negative, or suspect can be determined based on a minimum number of positive wells or by using a statistical protocol based on early (pre-inflection) reads for that sample and control samples tested on the same plate [36,37].

Controls and limitations in RT-QuIC assays are those common to all laboratory assays. One of the most important is the preparation and quality control of the recombinant PrP substrate (rPrP)—which is essential to ensuring sufficient assay sensitivity and specificity [36,39]. The rPrP auto-polymerization can be triggered by components in complex biologic samples (e.g., feces) and is a concern that requires sample-specific negative and positive controls. Another potential limitation is the presence of inhibitors of rPrP seeding in some samples, some of which can be managed by protocol modifications to overcome inhibition and/or enrichment for prions [18,40,41]. It is important that RT-QuIC assays include test-sample-matched negative and positive controls when assessing results.

The advantages of RT-QuIC are its relative rapidity compared with other prion assays, high sensitivity [36,38], adaptability to complex antemortem samples such as blood, saliva, excreta, and other tissues [39,40,42,43,44,45,46,47,48,49], and scalability. Serial tonsil/rectal biopsy studies in deer have shown that RT-QuIC can detect the onset of CWD infection weeks before immunohistochemistry (IHC), such that RT-QuIC positivity predicts subsequent positivity by IHC [50,51]. Because antemortem samples such as body fluids and excreta contain prion levels that are several logs lower than those in prime target tissues (e.g., brain, lymphoid tissue) [47], at present, RT-QuIC protocols based on these samples are performed chiefly in research studies of CWD. However, appropriately adapted RT-QuIC assays are now approved and widely used for the diagnosis of Creutzfeldt-Jakob Disease in humans; cerebrospinal fluid is most commonly used as a biospecimen, but other samples, including nasal brushings and skin, have shown promise as well [52,53]. The RT-QuIC assay format with appropriate substrates is now also being used to sensitively detect abnormal protein aggregates associated with Parkinson’s [54,55,56], Alzheimer’s, and related diseases [57,58,59].

## 4. The Process for CWD Diagnostic Assay Approval by the USDA

What: An official CWD test according to the USDA is defined as follows: (i) histopathological examination of central nervous system (CNS) tissues from the animal for characteristic microscopic lesions of CWD, using test protocols provided by the National Veterinary Services Laboratories (NVSL); (ii) the use of proteinase-resistant protein analysis methods including but not limited to IHC and/or western blotting on CNS and/or peripheral tissue samples from a live or dead animal, using test protocols provided by NVSL; or (iii) any other test method approved by the Administrator. It is the approval of a new test method that is of interest here.

Why: Diagnostic techniques are evolving, and the creation of new methods or applications for detecting CWD prions or diagnosing CWD could enhance CWD research and management. Obtaining official approval for a novel CWD diagnostic assay through the USDA is important for several reasons. First, it ensures that the characteristics of the assay are well established so assay results can be properly interpreted relative to the likelihood of the presence or absence of CWD/prions given the test result. The approval process also ensures that there is a standardized protocol for implementing the assay and that the results are repeatable within and across laboratories. This provides the foundation for official decision-making based on the assay’s results.

How: The approval process for a diagnostic assay to be utilized in an official capacity by a USDA CWD program varies depending on the type of assay under consideration. If the proposed assay is put forth as a test kit, the process can be found at https://www.aphis.usda.gov/animal_health/vet_biologics/publications/memo800-73.pdf (accessed on 30 January 2024).

If an assay is not part of a commercial test kit, then the process can be dependent upon the requirements outlined in the relevant USDA Animal and Plant Health Inspection Service (APHIS) disease program rules. Official testing for CWD is outlined in Title 9 Code of Federal Register (9CFR) Part 55.8 titled “Official CWD tests and approval of laboratories to conduct Official CWD tests” www.ecfr.gov/current/title-9/chapter-I/subchapter-B/part-55/subpart-A/section-55.8 (accessed on 30 January 2024). In short, the following information is required for approval: (i) a standardized test protocol is developed that must include a description of the test, a description of the reagents, materials, and equipment used for the test, the test methodology, and any control or quality assurance procedures; (ii) data exist to support reproducibility, that is, the ability to reproduce the same result repeatedly on a given sample; (iii) data to support suitability, that is, data to show that similar results can be produced when the test is run at other laboratories; (iv) data to support the sensitivity and specificity of the test; and (v) any other data requested by the Administrator to determine the suitability of the test for program use.

Who: The USDA is responsible for approving new tests for official diagnosis of CWD conducted on live or dead animals and will base their approval or disapproval of a test on the evaluation conducted by the APHIS and, when appropriate, outside scientists.

When: The timeline for approval varies depending on the diagnostic assay. However, collecting the information required for APHIS evaluation with sufficient statistical rigor is time-consuming and, therefore, it is common for the approval process to take 3–5 years.

## 5. CWD in the Environment

**Figure 3:**
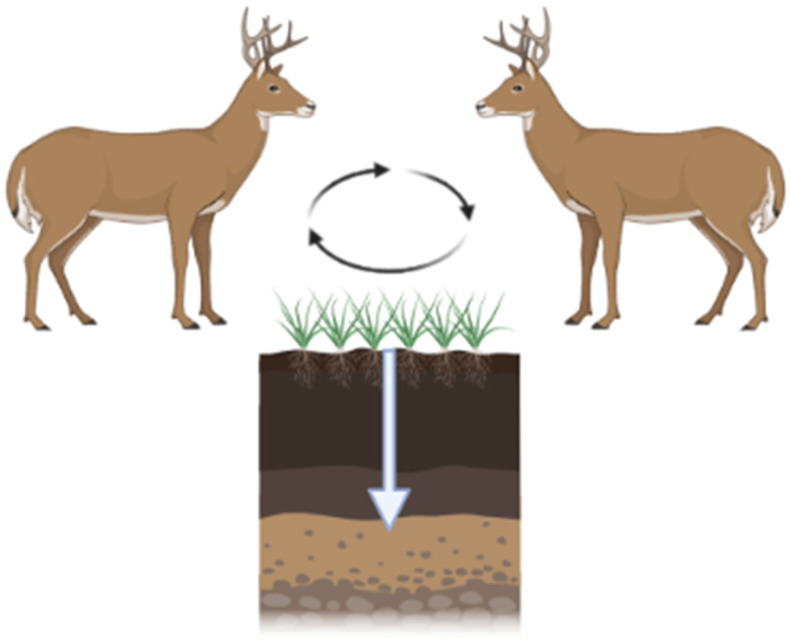
CWD prions are shed by infected hosts via urine, feces, and saliva. These prions are maintained in the environment by binding to vegetation and/or soil. Ingestion of soil/vegetation results in further transmission. Black arrows depict CWD prion cycling between hosts and the environment. White arrow depicts CWD prion migration through soils. Figure generated by BioRender.

CWD is increasing in prevalence and expanding its geographic range. The modes of transmission include direct animal-to-animal and indirect via the environment. Infected animals can contaminate their environment by shedding CWD prions throughout the incubation period (2+ years from the time of infection; Figure 3). CWD prions are shed in urine, feces, antler velvet, saliva, and, ultimately, carcasses [60,61,62]. Additionally, birthing fluids and tissues may also be sources of contamination at birth sites given CWD prion detection within the pregnancy microenvironment [63]. Shed prions bind to soils and soil minerals [64], plant materials [26], and other environmental surfaces [22]. Due to the extreme resistance of prions to physicochemical degradation, CWD prions can remain in the environment for extended periods of time.

The persistence of prions was first demonstrated experimentally by Russell Greig, who demonstrated that scrapie can be transmitted via contaminated pastures [65]. Brown and Gajdusek [66] showed that hamster prions, buried with soil in clay pots, remained infectious for >3 years, and subsequent work with BSE prions indicated that BSE infectivity was maintained, in burial pits, for at least 5 years [67]. Scrapie prions persisted in a contaminated sheep-house for at least 16 years [68]. Less is known about the persistence of CWD prions on surfaces (i.e., not buried samples) but infection of naïve deer introduced to highly contaminated pens indicates infectivity is retained for at least 5 years [69].

Prions can bind avidly to soil and soil minerals [64]. Binding to some soil types results in both increased disease penetrance and infectivity. Avidity of binding and migration of the prions through soil horizons is soil-type dependent. Clay-rich soils bind prions tightly, reducing the movement of the prions through the soil [70,71,72,73]. Soils rich in quartz sands, conversely, bind the prions less avidly and the prions readily migrate through the soil column. In experimental soil columns, prions can be detected in the leachate from the quartz-rich soils [74]. The avid binding and lack of migration of prions through clay soils have resulted in the recommendation of clay liners for carcass disposal pits [75]. Precipitation and freezing/thawing of soil can reduce infectivity [76].

CWD prions shed into the environment may also bind to vegetation. PMCA analysis of spiked grass plants indicates that CWD prions bind to the surface of plants, remaining bioavailable [26]. Experimentally, it has been demonstrated that prions can enter plants via their root system and migrate to the aerial portions of the plant [26,77].

Serial PMCA studies indicate that CWD prions are also present in environmental water sources, both in streams and in water treatment plants [78]. Prion levels in these studies are, however, below infectious doses.

Contaminated environments serve as reservoirs of CWD infection, a risk for both intra- and inter-species transmission of CWD; animals that consume soil/plant material from CWD-endemic areas are exposed to increasingly larger doses of CWD prions. The amount of CWD infectivity present in various environments has not been empirically determined. CWD prions are not evenly distributed across the landscape but are associated with specific sites where cervid activity is highest. Plummer et al., for example, detected CWD prions in mineral licks [23]. Ongoing research is measuring infectivity in highly endemic regions. A caveat to determining CWD prion persistence is that the ability of current rapid in vitro assays to detect CWD in soil declines with time, although there is no concomitant decrease in infectivity [79].

Remediation of contaminated environments currently depends on the removal of topsoil and/or treatment of the environment with 1N NaOH [80]. These treatments reduce prion infectivity slightly but are not practical for decontamination of the large areas currently affected by CWD. Other remediation approaches, including the use of humic acid, an organic component of many soil types [81], Prionzyme [28], and incineration [82] reduce prion infectivity in experimental studies. The development of remediation approaches is dependent on the detection of CWD in environmental samples and measurements of biologically relevant levels of contamination.

In summary, the contamination of the environment by CWD-affected cervids results in a long-term reservoir of prion infectivity. Management strategies will benefit from the inclusion of environmental decontamination approaches.

## 6. Interspecies Transmission of CWD

As previously mentioned, prions can transmit between species (i.e., interspecies transmission) in both experimental and natural settings. For example, bovine spongiform encephalopathy (BSE, a.k.a. mad cow disease) can be transmitted to numerous species, including humans, in experimental and/or natural settings [83,84,85,86,87,88,89]. This is in stark contrast to scrapie in sheep and goats which does not readily infect other species in natural settings and therefore exhibits strong species barriers [90]. As numerous, largely unquantifiable, factors could influence relative interspecies transmission efficiencies via natural routes of exposure, the risks of interspecies transmissions must be approximated experimentally.

**Figure 4:**
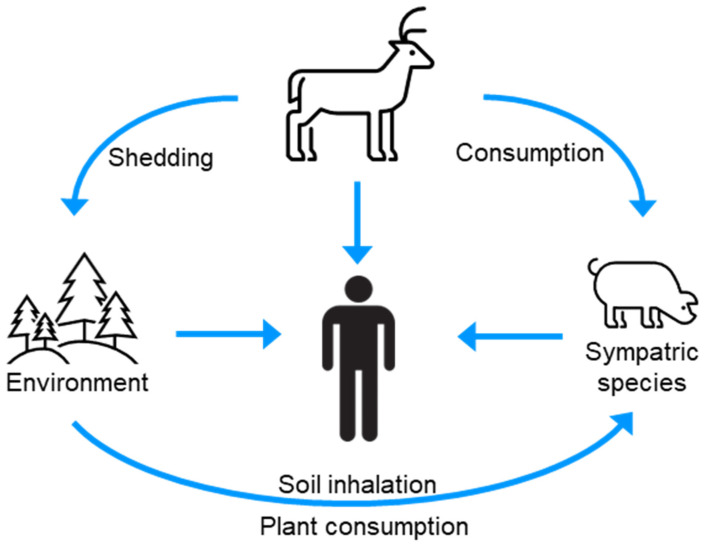
The infection cycle indicates sources of CWD for interspecies transmission.

Numerous species, including humans, are exposed to CWD prions via contact with or consumption of infected animals, or CWD-contaminated environments (Figure 4) [91]. The host range of CWD is poorly defined; however, laboratory studies have provided evidence for the species barrier of CWD prions for several sympatric species. The interpretation of these studies is, however, nuanced and warrants consideration in the broader context of the prion literature.

Experimental animal transmission studies have not provided a clear picture of the zoonotic potential of CWD prions. Transmission of BSE to macaques, a non-human primate, provided compelling evidence that the emergence of variant Creutzfeldt-Jakob (vCJD) disease in humans was the result of interspecies transmission of BSE [85,92]. Macaques are susceptible to BSE and CJD infections, two prions that are known to infect humans [93]. Macaques are also susceptible to scrapie; however, compared to BSE, the incubation period is twice as long (6–10 years), with an incomplete attack rate consistent with a historical lack of association of scrapie with human prion disease [94]. Two separate independent experimental infection studies of macaques with CWD resulted in different outcomes. In one study, inoculation of macaques with CWD failed to cause clinical disease or evidence of a subclinical infection out to 13 years post-infection (the National Institutes of Health study) [95,96,97]. In a second unpublished study, transmission of CWD to macaques also failed to cause clinical disease, but, in a subset of animals, evidence for a subclinical infection was observed (the Canada study). Because the Canada study has not been published, it is impossible to determine whether the different outcomes are due to differences in the strains of CWD agents used or methods of inoculation. Because the species barrier is largely governed by differences between the agent and host PrP, and macaque and human PrP^C^ differ by nine amino acids, transmission, or lack thereof, may not provide conclusive evidence for the zoonotic potential of CWD. To match the PrP^C^ sequence with human PrP^C^, transgenic mice that express human PrP^C^ have been inoculated with CWD. To date, CWD has largely failed to cause disease in these mice [98,99,100,101,102,103]; however, a recent study provides evidence that CWD can infect transgenic mice that express human PrP^C^, albeit with a low attack rate and at times post-infection that are at the end of the animal’s life span [104]. CWD can infect other sympatric species following direct inoculation of the brain (e.g., cattle, cats); however, these species do not develop disease following natural routes of infection [105,106,107,108,109,110,111,112,113]. Importantly, swine and sheep are an exception where ingestion or nasal exposure of CWD prions results in replication of CWD prions, but these animals do not develop clinical signs of disease [114,115].

The above transmission studies warrant interpretation with caution for the following reasons: First, the animals used do not fully recapitulate humans. Non-human primates have different PrP sequences, and if host factors in addition to PrP are involved, these are not accounted for [116,117,118]. Mice have a relatively short life span that may not allow sufficient time for disease to develop [119]. Second, prion strains in the same host species can have a different capacity to infect new species [120,121]. Multiple strains of CWD exist; however, the potential of these strains to infect other species is unknown [101,122,123,124,125,126,127,128,129,130,131,132]. Third, the binding of prions to particles (e.g., soil) and the number and route of exposures of a host to prions can profoundly affect infection; this has not been fully examined for CWD [64,70,133,134,135,136]. Fourth, interspecies transmission of prions can expand their host range [113,137]. If CWD infects a sympatric species (e.g., swine), the host range of the new species will have to be empirically determined. To address these limitations, methods for assessing the host range of prions in vitro have been developed.

Biochemical assays have also investigated the species barrier of CWD prions. Cell-free conversion is an in vitro prion amplification technique that recapitulates the known host range of prions [5]. These studies found that, while CWD prions can convert human PrP^C^ to PrP^Sc^, the efficiency of this conversion is like scrapie conversion of human PrP^C^ to PrP^Sc^ indicating that a high species barrier exists between CWD and humans [138,139]. As previously described, PMCA, an in vitro technique that replicates infectious prions, has been used to examine the species barrier effect [14,140,141,142,143]; however, PMCA is sufficiently powerful that it can overcome known natural species barriers (e.g., rabbits), and studies where CWD prions can convert human PrP^C^ to infectious PrP^Sc^ warrant interpretation with much caution [31,144,145].

Overall, experimental evidence indicates that CWD prions cannot readily cross species barriers. However, risk continues to increase as both the geographic range and prevalence of CWD-infected animals increase. Like any pathogen, increased CWD prevalence and prion replication provide opportunities for adaptations, mutations, and zoonoses. Many important variables known to influence the species barrier effect have not been tested. Because prion diseases are 100% fatal with no known treatment or therapy, an abundance of caution is warranted.

## 7. Summary

As outlined above, there have been marked improvements in methods for sensitive and specific detection of CWD prions (PMCA and RT-QuIC). As these methods continue to be refined, they will allow for improved detection of CWD in live animals, providing additional tools for the management and mitigation of CWD. Complicating management, however, is the persistence of CWD prions in the environment. Consortium members are involved in several research projects aimed at determining the parameters of persistence, optimizing detection methods in complicated environmental matrices, and developing methods for decontamination. The zoonotic potential of CWD is not yet clear, and data indicate that the species barrier is high but perhaps not impenetrable. Although the state of the science in CWD research described herein is not all-encompassing, the combination of these factors alone highlights the complexity of CWD as a multi-faceted health problem. It is the goal of the consortium to combine research and management efforts across North America to address these issues, with the long-term goal of reducing, if not mitigating, CWD.

## References

[1] Williams E.S., Young S. (1980). Chronic wasting disease of captive mule deer: A spongiform encephalopathy. J. Wildl. Dis..

[2] Deleault N.R., Harris B.T., Rees J.R., Supattapone S. (2007). Formation of native prions from minimal components in vitro. Proc. Natl. Acad. Sci. USA.

[3] Prusiner S.B. (1982). Novel proteinaceous infectious particles cause scrapie. Science.

[4] Kraus A., Hoyt F., Schwartz C.L., Hansen B., Artikis E., Hughson A.G., Raymond G.J., Race B., Baron G.S., Caughey B. (2021). High-resolution structure and strain comparison of infectious mammalian prions. Mol. Cell.

[5] Kocisko D.A., Come J.H., Priola S.A., Chesebro B., Raymond G.J., Lansbury P.T., Caughey B. (1994). Cell-free formation of protease-resistant prion protein. Nature.

[6] Oesch B., Westaway D., Walchli M., McKinley M.P., Kent S.B., Aebersold R., Barry R.A., Tempst P., Teplow D.B., Hood L.E. (1985). A cellular gene encodes scrapie PrP 27-30 protein. Cell.

[7] Caughey B., Raymond G.J. (1991). The scrapie-associated form of PrP is made from a cell surface precursor that is both protease- and phospholipase-sensitive. J. Biol. Chem..

[8] Otero A., Velasquez C.D., Aiken J., McKenzie D. (2021). Chronic wasting disease: A cervid prion infection looming to spillover. Vet. Res..

[9] Brandel J.P., Culeux A., Grznarova K., Levavasseur E., Lamy P., Privat N., Welaratne A., Denouel A., Laplanche J.L., Haik S. (2019). Amplification techniques and diagnosis of prion diseases. Rev. Neurol..

[10] Haley N.J., Richt J.A. (2017). Evolution of diagnostic tests for chronic wasting eisease, a naturally occurring prion disease of cervids. Pathogens.

[11] Morales R., Duran-Aniotz C., Diaz-Espinoza R., Camacho M.V., Soto C. (2012). Protein misfolding cyclic amplification of infectious prions. Nat. Protoc..

[12] Schmitz M., Cramm M., Llorens F., Muller-Cramm D., Collins S., Atarashi R., Satoh K., Orru C.D., Groveman B.R., Zafar S. (2016). The real-time quaking-induced conversion assay for detection of human prion disease and study of other protein misfolding diseases. Nat. Protoc..

[13] Castilla J., Saa P., Hetz C., Soto C. (2005). In vitro generation of infectious scrapie prions. Cell.

[14] Saborio G.P., Permanne B., Soto C. (2001). Sensitive detection of pathological prion protein by cyclic amplification of protein misfolding. Nature.

[15] Saa P., Castilla J., Soto C. (2006). Ultra-efficient replication of infectious prions by automated protein misfolding cyclic amplification. J. Biol. Chem..

[16] Chen B., Morales R., Barria M.A., Soto C. (2010). Estimating prion concentration in fluids and tissues by quantitative PMCA. Nat. Methods.

[17] McNulty E., Nalls A.V., Mellentine S., Hughes E., Pulscher L., Hoover E.A., Mathiason C.K. (2019). Comparison of conventional, amplification and bio-assay detection methods for a chronic wasting disease inoculum pool. PLoS ONE.

[18] Davenport K.A., Hoover C.E., Denkers N.D., Mathiason C.K., Hoover E.A. (2018). Modified protein misfolding cyclic amplification overcomes real-time quaking-induced conversion assay inhibitors in deer saliva to detect chronic wasting disease prions. J. Clin. Microbiol..

[19] Kramm C., Soto P., Nichols T.A., Morales R. (2020). Chronic wasting disease (CWD) prion detection in blood from pre-symptomatic white-tailed deer harboring PRNP polymorphic variants. Sci. Rep..

[20] Nalls A.V., McNulty E.E., Mayfield A., Crum J.M., Keel M.K., Hoover E.A., Ruder M.G., Mathiason C.K. (2021). Detection of chronic wasting disease prions in fetal tissues of free-ranging white-tailed deer. Viruses.

[21] Bravo-Risi F., Soto P., Eckland T., Dittmar R., Ramirez S., Catumbela C.S.G., Soto C., Lockwood M., Nichols T., Morales R. (2021). Detection of CWD prions in naturally infected white-tailed deer fetuses and gestational tissues by PMCA. Sci. Rep..

[22] Pritzkow S., Morales R., Lyon A., Concha-Marambio L., Urayama A., Soto C. (2018). Efficient prion disease transmission through common environmental materials. J. Biol. Chem..

[23] Plummer I.H., Johnson C.J., Chesney A.R., Pedersen J.A., Samuel M.D. (2018). Mineral licks as environmental reservoirs of chronic wasting disease prions. PLoS ONE.

[24] Haley N.J., Mathiason C.K., Zabel M.D., Telling G.C., Hoover E.A. (2009). Detection of sub-clinical CWD infection in conventional test-negative deer long after oral exposure to urine and feces from CWD+ deer. PLoS ONE.

[25] Johnson C.J., Aiken J.M., McKenzie D., Samuel M.D., Pedersen J.A. (2012). Highly efficient amplification of chronic wasting disease agent by protein misfolding cyclic amplification with beads (PMCAb). PLoS ONE.

[26] Pritzkow S., Morales R., Moda F., Khan U., Telling G.C., Hoover E., Soto C. (2015). Grass plants bind, retain, uptake, and transport infectious prions. Cell Rep..

[27] Saunders S.E., Shikiya R.A., Langenfeld K., Bartelt-Hunt S.L., Bartz J.C. (2011). Replication efficiency of soil-bound prions varies with soil type. J. Virol..

[28] Saunders S.E., Bartz J.C., Vercauteren K.C., Bartelt-Hunt S.L. (2011). An enzymatic treatment of soil-bound prions effectively inhibits replication. Appl. Environ. Microbiol..

[29] Kramm C., Gomez-Gutierrez R., Soto C., Telling G., Nichols T., Morales R. (2019). In Vitro detection of Chronic Wasting Disease (CWD) prions in semen and reproductive tissues of white-tailed deer bucks (*Odocoileus virginianus*). PLoS ONE.

[30] Nalls A.V., McNulty E., Powers J., Seelig D.M., Hoover C., Haley N.J., Hayes-Klug J., Anderson K., Stewart P., Goldmann W. (2013). Mother to offspring transmission of chronic wasting disease in reeves’ muntjac deer. PLoS ONE.

[31] Barria M.A., Telling G.C., Gambetti P., Mastrianni J.A., Soto C. (2011). Generation of a new form of human PrP(Sc) in vitro by interspecies transmission from cervid prions. J. Biol. Chem..

[32] Castilla J., Gonzalez-Romero D., Saa P., Morales R., De Castro J., Soto C. (2008). Crossing the species barrier by PrP(Sc) replication in vitro generates unique infectious prions. Cell.

[33] Green K.M., Castilla J., Seward T.S., Napier D.L., Jewell J.E., Soto C., Telling G.C. (2008). Accelerated high fidelity prion amplification within and across prion species barriers. PLoS Pathog..

[34] Morales R. (2017). Prion strains in mammals: Different conformations leading to disease. PLoS Pathog..

[35] Morales R., Abid K., Soto C. (2007). The prion strain phenomenon: Molecular basis and unprecedented features. Biochim. Biophys. Acta.

[36] Wilham J.M., Orru C.D., Bessen R.A., Atarashi R., Sano K., Race B., Meade-White K.D., Taubner L.M., Timmes A., Caughey B. (2010). Rapid end-point quantitation of prion seeding activity with sensitivity comparable to bioassays. PLoS Pathog..

[37] Atarashi R., Satoh K., Sano K., Fuse T., Yamaguchi N., Ishibashi D., Matsubara T., Nakagaki T., Yamanaka H., Shirabe S. (2011). Ultrasensitive human prion detection in cerebrospinal fluid by real-time quaking-induced conversion. Nat. Med..

[38] Henderson D.M., Davenport K.A., Haley N.J., Denkers N.D., Mathiason C.K., Hoover E.A. (2015). Quantitative assessment of prion infectivity in tissues and body fluids by real-time quaking-induced conversion. J. Gen. Virol..

[39] Orru C.D., Wilham J.M., Vascellari S., Hughson A.G., Caughey B. (2012). New generation QuIC assays for prion seeding activity. Prion.

[40] Orru C.D., Wilham J.M., Raymond L.D., Kuhn F., Schroeder B., Raeber A.J., Caughey B. (2011). Prion disease blood test using immunoprecipitation and improved quaking-induced conversion. mBio.

[41] Denkers N.D., Henderson D.M., Mathiason C.K., Hoover E.A. (2016). Enhanced prion detection in biological samples by magnetic particle extraction and real-time quaking-induced conversion. J. Gen. Virol..

[42] Elder A.M., Henderson D.M., Nalls A.V., Hoover E.A., Kincaid A.E., Bartz J.C., Mathiason C.K. (2015). Immediate and Ongoing Detection of Prions in the Blood of Hamsters and Deer following Oral, Nasal, or Blood Inoculations. J. Virol..

[43] Elder A.M., Henderson D.M., Nalls A.V., Wilham J.M., Caughey B.W., Hoover E.A., Kincaid A.E., Bartz J.C., Mathiason C.K. (2013). In vitro detection of prionemia in TSE-infected cervids and hamsters. PLoS ONE.

[44] Haley N.J., Van de Motter A., Carver S., Henderson D., Davenport K., Seelig D.M., Mathiason C., Hoover E. (2013). Prion-seeding activity in cerebrospinal fluid of deer with chronic wasting disease. PLoS ONE.

[45] Henderson D.M., Denkers N.D., Hoover C.E., Garbino N., Mathiason C.K., Hoover E.A. (2015). Longitudinal detection of prion shedding in saliva and urine by chronic wasting disease-infected deer by real-time quaking-induced conversion. J. Virol..

[46] Tennant J.M., Li M., Henderson D.M., Tyer M.L., Denkers N.D., Haley N.J., Mathiason C.K., Hoover E.A. (2020). Shedding and stability of CWD prion seeding activity in cervid feces. PLoS ONE.

[47] Cooper S.K., Hoover C.E., Henderson D.M., Haley N.J., Mathiason C.K., Hoover E.A. (2019). Detection of CWD in cervids by RT-QuIC assay of third eyelids. PLoS ONE.

[48] Ferreira N.C., Charco J.M., Plagenz J., Orru C.D., Denkers N.D., Metrick M.A., Hughson A.G., Griffin K.A., Race B., Hoover E.A. (2021). Detection of chronic wasting disease in mule and white-tailed deer by RT-QuIC analysis of outer ear. Sci. Rep..

[49] Henderson D.M., Tennant J.M., Haley N.J., Denkers N.D., Mathiason C.K., Hoover E.A. (2017). Detection of chronic wasting disease prion seeding activity in deer and elk feces by real-time quaking-induced conversion. J. Gen. Virol..

[50] Henderson D.M., Denkers N.D., Hoover C.E., McNulty E.E., Cooper S.K., Bracchi L.A., Mathiason C.K., Hoover E.A. (2020). Progression of chronic wasting disease in white-tailed deer analyzed by serial biopsy RT-QuIC and immunohistochemistry. PLoS ONE.

[51] Denkers N.D., Hoover C.E., Davenport K.A., Henderson D.M., McNulty E.E., Nalls A.V., Mathiason C.K., Hoover E.A. (2020). Very low oral exposure to prions of brain or saliva origin can transmit chronic wasting disease. PLoS ONE.

[52] Orru C.D., Groveman B.R., Hughson A.G., Zanusso G., Coulthart M.B., Caughey B. (2015). Rapid and sensitive RT-QuIC detection of human Creutzfeldt-Jakob disease using cerebrospinal fluid. mBio.

[53] Vascellari S., Orru C.D., Caughey B. (2022). Real-Time Quaking- Induced Conversion Assays for Prion Diseases, Synucleinopathies, and Tauopathies. Front. Aging Neurosci..

[54] Fairfoul G., McGuire L.I., Pal S., Ironside J.W., Neumann J., Christie S., Joachim C., Esiri M., Evetts S.G., Rolinski M. (2016). Alpha-synuclein RT-QuIC in the CSF of patients with alpha-synucleinopathies. Ann. Clin. Transl. Neurol..

[55] Groveman B.R., Orru C.D., Hughson A.G., Raymond L.D., Zanusso G., Ghetti B., Campbell K.J., Safar J., Galasko D., Caughey B. (2018). Rapid and ultra-sensitive quantitation of disease-associated alpha-synuclein seeds in brain and cerebrospinal fluid by alphaSyn RT-QuIC. Acta Neuropathol. Commun..

[56] Russo M.J., Orru C.D., Concha-Marambio L., Giaisi S., Groveman B.R., Farris C.M., Holguin B., Hughson A.G., LaFontant D.E., Caspell-Garcia C. (2021). High diagnostic performance of independent alpha-synuclein seed amplification assays for detection of early Parkinson’s disease. Acta Neuropathol. Commun..

[57] Saijo E., Ghetti B., Zanusso G., Oblak A., Furman J.L., Diamond M.I., Kraus A., Caughey B. (2017). Ultrasensitive and selective detection of 3-repeat tau seeding activity in Pick disease brain and cerebrospinal fluid. Acta Neuropathol..

[58] Kraus A., Saijo E., Metrick M.A., Newell K., Sigurdson C.J., Zanusso G., Ghetti B., Caughey B. (2019). Seeding selectivity and ultrasensitive detection of tau aggregate conformers of Alzheimer disease. Acta Neuropathol..

[59] Metrick M.A., Ferreira N.D.C., Saijo E., Kraus A., Newell K., Zanusso G., Vendruscolo M., Ghetti B., Caughey B. (2020). A single ultrasensitive assay for detection and discrimination of tau aggregates of Alzheimer and Pick diseases. Acta Neuropathol. Commun..

[60] Mathiason C.K., Powers J.G., Dahmes S.J., Osborn D.A., Miller K.V., Warren R.J., Mason G.L., Hays S.A., Hayes-Klug J., Seelig D.M. (2006). Infectious prions in the saliva and blood of deer with chronic wasting disease. Science.

[61] Tamguney G., Miller M.W., Wolfe L.L., Sirochman T.M., Glidden D.V., Palmer C., Lemus A., DeArmond S.J., Prusiner S.B. (2009). Asymptomatic deer excrete infectious prions in faeces. Nature.

[62] Angers R.C., Seward T.S., Napier D., Green M., Hoover E., Spraker T., O’Rourke K., Balachandran A., Telling G.C. (2009). Chronic wasting disease prions in elk antler velvet. Emerg. Infect. Dis..

[63] Nalls A.V., McNulty E., Hoover C.E., Pulscher L.A., Hoover E.A., Mathiason C.K. (2017). Infectious prions in the pregnancy microenvironment of chronic wasting disease-infected Reeves’ muntjac deer. J. Virol..

[64] Johnson C.J., Pedersen J.A., Chappell R.J., McKenzie D., Aiken J.M. (2007). Oral transmissibility of prion disease is enhanced by binding to soil particles. PLoS Pathog..

[65] Russell Greig J. (1940). Observations on the Transmission of the Disease by Mediate Contact. Vet. J..

[66] Brown P., Gajdusek D.C. (1991). Survival of scrapie virus after 3 years’ interment. Lancet.

[67] Somerville R.A., Fernie K., Smith A., Bishop K., Maddison B.C., Gough K.C., Hunter N. (2019). BSE infectivity survives burial for five years with only limited spread. Arch. Virol..

[68] Georgsson G., Sigurdarson S., Brown P. (2006). Infectious agent of sheep scrapie may persist in the environment for at least 16 years. J. Gen. Virol..

[69] Miller M.W., Williams E.S., Hobbs N.T., Wolfe L.L. (2004). Environmental sources of prion transmission in mule deer. Emerg. Infect. Dis..

[70] Johnson C.J., Phillips K.E., Schramm P.T., McKenzie D., Aiken J.M., Pedersen J.A. (2006). Prions adhere to soil minerals and remain infectious. PLoS Pathog..

[71] Giachin G., Narkiewicz J., Scaini D., Ngoc A.T., Margon A., Sequi P., Leita L., Legname G. (2014). Prion protein interaction with soil humic substances: Environmental implications. PLoS ONE.

[72] Saunders S.E., Bartelt-Hunt S.L., Bartz J.C. (2012). Resistance of soil-bound prions to rumen digestion. PLoS ONE.

[73] David Walter W., Walsh D.P., Farnsworth M.L., Winkelman D.L., Miller M.W. (2011). Soil clay content underlies prion infection odds. Nat. Commun..

[74] Kuznetsova A., McKenzie D., Ytrehus B., Utaaker K.S., Aiken J.M. (2023). Movement of chronic wasting disease prions in prairie, boreal and alpine soils. Pathogens.

[75] Jacobson K.H., Lee S., McKenzie D., Benson C.H., Pedersen J.A. (2009). Transport of the pathogenic prion protein through landfill materials. Environ. Sci. Technol..

[76] Yuan Q., Telling G., Bartelt-Hunt S.L., Bartz J.C. (2018). Dehydration of Prions on environmentally relevant surfaces protects them from inactivation by freezing and thawing. J. Virol..

[77] Carlson C.M., Thomas S., Keating M.W., Soto P., Gibbs N.M., Chang H., Wiepz J.K., Austin A.G., Schneider J.R., Morales R. (2023). Plants as vectors for environmental prion transmission. iScience.

[78] Nichols T.A., Pulford B., Wyckoff A.C., Meyerett C., Michel B., Gertig K., Hoover E.A., Jewell J.E., Telling G.C., Zabel M.D. (2009). Detection of protease-resistant cervid prion protein in water from a CWD-endemic area. Prion.

[79] Kuznetsova A., McKenzie D., Cullingham C., Aiken J.M. (2020). Long-term incubation PrP(CWD) with soils affects prion recovery but not Infectivity. Pathogens.

[80] Sohn H.J., Park K.J., Roh I.S., Kim H.J., Park H.C., Kang H.E. (2019). Sodium hydroxide treatment effectively inhibits PrP(CWD) replication in farm soil. Prion.

[81] Kuznetsova A., Cullingham C., McKenzie D., Aiken J.M. (2018). Soil humic acids degrade CWD prions and reduce infectivity. PLoS Pathog..

[82] Brown P., Rau E.H., Lemieux P., Johnson B.K., Bacote A.E., Gajdusek D.C. (2004). Infectivity studies of both ash and air emissions from simulated incineration of scrapie-contaminated tissues. Environ. Sci. Technol..

[83] Bruce M.E., Will R.G., Ironside J.W., McConnell I., Drummond D., Suttie A., McCardle L., Chree A., Hope J., Birkett C. (1997). Transmissions to mice indicate that ‘new variant’ CJD is caused by the BSE agent. Nature.

[84] Dagleish M.P., Martin S., Steele P., Finlayson J., Siso S., Hamilton S., Chianini F., Reid H.W., Gonzalez L., Jeffrey M. (2008). Experimental transmission of bovine spongiform encephalopathy to European red deer (*Cervus elaphus elaphus*). BMC Vet. Res..

[85] Lasmezas C.I., Deslys J.P., Demaimay R., Adjou K.T., Lamoury F., Dormont D., Robain O., Ironside J., Hauw J.J. (1996). BSE transmission to macaques. Nature.

[86] Kirkwood J.K., Wells G.A., Wilesmith J.W., Cunningham A.A., Jackson S.I. (1990). Spongiform encephalopathy in an arabian oryx (*Oryx leucoryx*) and a greater kudu (*Tragelaphus strepsiceros*). Vet. Rec..

[87] Wells G.A. (2003). Pathogenesis of BSE. Vet. Res. Commun..

[88] Pearson G.R., Wyatt J.M., Gruffydd-Jones T.J., Hope J., Chong A., Higgins R.J., Scott A.C., Wells G.A. (1992). Feline spongiform encephalopathy: Fibril and PrP studies. Vet. Rec..

[89] Wyatt J.M., Pearson G.R., Smerdon T.N., Gruffydd-Jones T.J., Wells G.A., Wilesmith J.W. (1991). Naturally occurring scrapie-like spongiform encephalopathy in five domestic cats. Vet. Rec..

[90] Jaumain E., Quadrio I., Herzog L., Reine F., Rezaei H., Andreoletti O., Laude H., Perret-Liaudet A., Haik S., Beringue V. (2016). Absence of evidence for a causal link between bovine spongiform encephalopathy strain variant L-BSE and known forms of sporadic Creutzfeldt-Jakob disease in human PrP transgenic mice. J. Virol..

[91] Osterholm M.T., Anderson C.J., Zabel M.D., Scheftel J.M., Moore K.A., Appleby B.S. (2019). Chronic wasting disease in cervids: Implications for prion transmission to humans and other animal species. mBio.

[92] Lasmezas C.I., Fournier J.G., Nouvel V., Boe H., Marce D., Lamoury F., Kopp N., Hauw J.J., Ironside J., Bruce M. (2001). Adaptation of the bovine spongiform encephalopathy agent to primates and comparison with Creutzfeldt—Jakob disease: Implications for human health. Proc. Natl. Acad. Sci. USA.

[93] Mikol J., Delmotte J., Jouy D., Vaysset E., Bastian C., Deslys J.P., Comoy E. (2021). Direct neural transmission of vCJD/BSE in macaque after finger incision. Acta Neuropathol..

[94] Comoy E.E., Mikol J., Luccantoni-Freire S., Correia E., Lescoutra-Etchegaray N., Durand V., Dehen C., Andreoletti O., Casalone C., Richt J.A. (2015). Transmission of scrapie prions to primate after an extended silent incubation period. Sci. Rep..

[95] Race B., Meade-White K.D., Miller M.W., Barbian K.D., Rubenstein R., LaFauci G., Cervenakova L., Favara C., Gardner D., Long D. (2009). Susceptibilities of nonhuman primates to chronic wasting disease. Emerg. Infect. Dis..

[96] Race B., Meade-White K.D., Phillips K., Striebel J., Race R., Chesebro B. (2014). Chronic wasting disease agents in nonhuman primates. Emerg. Infect. Dis..

[97] Race B., Williams K., Orru C.D., Hughson A.G., Lubke L., Chesebro B. (2018). Lack of transmission of chronic wasting disease to Cynomolgus macaques. J. Virol..

[98] Race B., Williams K., Chesebro B. (2019). Transmission studies of chronic wasting disease to transgenic mice overexpressing human prion protein using the RT-QuIC assay. Vet. Res..

[99] Wilson R., Plinston C., Hunter N., Casalone C., Corona C., Tagliavini F., Suardi S., Ruggerone M., Moda F., Graziano S. (2012). Chronic wasting disease and atypical forms of bovine spongiform encephalopathy and scrapie are not transmissible to mice expressing wild-type levels of human prion protein. J. Gen. Virol..

[100] Sandberg M.K., Al-Doujaily H., Sigurdson C.J., Glatzel M., O’Malley C., Powell C., Asante E.A., Linehan J.M., Brandner S., Wadsworth J.D. (2010). Chronic wasting disease prions are not transmissible to transgenic mice overexpressing human prion protein. J. Gen. Virol..

[101] Tamguney G., Giles K., Bouzamondo-Bernstein E., Bosque P.J., Miller M.W., Safar J., DeArmond S.J., Prusiner S.B. (2006). Transmission of elk and deer prions to transgenic mice. J. Virol..

[102] Kong Q., Huang S., Zou W., Vanegas D., Wang M., Wu D., Yuan J., Zheng M., Bai H., Deng H. (2005). Chronic wasting disease of elk: Transmissibility to humans examined by transgenic mouse models. J. Neurosci..

[103] Kurt T.D., Jiang L., Fernandez-Borges N., Bett C., Liu J., Yang T., Spraker T.R., Castilla J., Eisenberg D., Kong Q. (2015). Human prion protein sequence elements impede cross-species chronic wasting disease transmission. J. Clin. Investig..

[104] Hannaoui S., Zemlyankina I., Chang S.C., Arifin M.I., Beringue V., McKenzie D., Schatzl H.M., Gilch S. (2022). Transmission of cervid prions to humanized mice demonstrates the zoonotic potential of CWD. Acta Neuropathol..

[105] Wolfe L.L., Fox K.A., Griffin K.A., Miller M.W. (2022). Mountain Lions (*Puma concolor*) resist long-term dietary exposure to chronic wasting disease. J. Wildl. Dis..

[106] Rhyan J.C., Miller M.W., Spraker T.R., McCollum M., Nol P., Wolfe L.L., Davis T.R., Creekmore L., O’Rourke K.I. (2011). Failure of fallow deer (*Dama dama*) to develop chronic wasting disease when exposed to a contaminated environment and infected mule deer (*Odocoileus hemionus*). J. Wildl. Dis..

[107] Williams E.S., O’Toole D., Miller M.W., Kreeger T.J., Jewell J.E. (2018). Cattle (*Bos taurus*) resist chronic wasting disease following oral inoculation challenge or ten years’ natural exposure in contaminated environments. J. Wildl. Dis..

[108] Mathiason C.K., Nalls A.V., Seelig D.M., Kraft S.L., Carnes K., Anderson K.R., Hayes-Klug J., Hoover E.A. (2013). Susceptibility of domestic cats to chronic wasting disease. J. Virol..

[109] Hamir A.N., Kunkle R.A., Cutlip R.C., Miller J.M., O’Rourke K.I., Williams E.S., Miller M.W., Stack M.J., Chaplin M.J., Richt J.A. (2005). Experimental transmission of chronic wasting disease agent from mule deer to cattle by the intracerebral route. J. Vet. Diagn. Investig..

[110] Hamir A.N., Kunkle R.A., Miller J.M., Greenlee J.J., Richt J.A. (2006). Experimental second passage of chronic wasting disease (CWD (mule deer)) agent to cattle. J. Comp. Pathol..

[111] Hamir A.N., Kunkle R.A., Cutlip R.C., Miller J.M., Williams E.S., Richt J.A. (2006). Transmission of chronic wasting disease of mule deer to Suffolk sheep following intracerebral inoculation. J. Vet. Diagn. Investig..

[112] Hamir A.N., Miller J.M., Kunkle R.A., Hall S.M., Richt J.A. (2007). Susceptibility of cattle to first-passage intracerebral inoculation with chronic wasting disease agent from white-tailed deer. Vet. Pathol..

[113] Bartz J.C., Marsh R.F., McKenzie D.I., Aiken J.M. (1998). The host range of chronic wasting disease is altered on passage in ferrets. Virology.

[114] Moore S.J., West Greenlee M.H., Kondru N., Manne S., Smith J.D., Kunkle R.A., Kanthasamy A., Greenlee J.J. (2017). Experimental transmission of the chronic wasting disease zgent to swine after oral or intracranial inoculation. J. Virol..

[115] Cassmann E.D., Greenlee J.J. (2020). Pathogenesis, detection, and control of scrapie in sheep. Am. J. Vet. Res..

[116] Saa P., Sferrazza G.F., Ottenberg G., Oelschlegel A.M., Dorsey K., Lasmezas C.I. (2012). Strain-specific role of RNAs in prion replication. J. Virol..

[117] Crowell J., Hughson A., Caughey B., Bessen R.A. (2015). Host Determinants of Prion Strain Diversity Independent of Prion Protein Genotype. J. Virol..

[118] Scott M., Foster D., Mirenda C., Serban D., Coufal F., Walchli M., Torchia M., Groth D., Carlson G., DeArmond S.J. (1989). Transgenic mice expressing hamster prion protein produce species-specific scrapie infectivity and amyloid plaques. Cell.

[119] Dickinson A.G., Fraser H., Outram G.W. (1975). Scrapie incubation time can exceed natural lifespan. Nature.

[120] Collinge J., Clarke A.R. (2007). A general model of prion strains and their pathogenicity. Science.

[121] Marin-Moreno A., Huor A., Espinosa J.C., Douet J.Y., Aguilar-Calvo P., Aron N., Piquer J., Lugan S., Lorenzo P., Tillier C. (2020). Radical Change in Zoonotic Abilities of Atypical BSE Prion Strains as Evidenced by Crossing of Sheep Species Barrier in Transgenic Mice. Emerg. Infect. Dis..

[122] Herbst A., Velasquez C.D., Triscott E., Aiken J.M., McKenzie D. (2017). Chronic Wasting Disease Prion Strain Emergence and Host Range Expansion. Emerg. Infect. Dis..

[123] Duque Velasquez C., Kim C., Haldiman T., Kim C., Herbst A., Aiken J., Safar J.G., McKenzie D. (2020). Chronic wasting disease (CWD) prion strains evolve via adaptive diversification of conformers in hosts expressing prion protein polymorphisms. J. Biol. Chem..

[124] Hannaoui S., Triscott E., Duque Velasquez C., Chang S.C., Arifin M.I., Zemlyankina I., Tang X., Bollinger T., Wille H., McKenzie D. (2021). New and distinct chronic wasting disease strains associated with cervid polymorphism at codon 116 of the Prnp gene. PLoS Pathog..

[125] Johnson C.J., Herbst A., Duque-Velasquez C., Vanderloo J.P., Bochsler P., Chappell R., McKenzie D. (2011). Prion protein polymorphisms affect chronic wasting disease progression. PLoS ONE.

[126] Bian J., Christiansen J.R., Moreno J.A., Kane S.J., Khaychuk V., Gallegos J., Kim S., Telling G.C. (2019). Primary structural differences at residue 226 of deer and elk PrP dictate selection of distinct CWD prion strains in gene-targeted mice. Proc. Natl. Acad. Sci. USA.

[127] Perrott M.R., Sigurdson C.J., Mason G.L., Hoover E.A. (2012). Evidence for distinct chronic wasting disease (CWD) strains in experimental CWD in ferrets. J. Gen. Virol..

[128] Nonno R., Di Bari M.A., Pirisinu L., D’Agostino C., Vanni I., Chiappini B., Marcon S., Riccardi G., Tran L., Vikoren T. (2020). Studies in bank voles reveal strain differences between chronic wasting disease prions from Norway and North America. Proc. Natl. Acad. Sci. USA.

[129] Bian J., Kim S., Kane S.J., Crowell J., Sun J.L., Christiansen J., Saijo E., Moreno J.A., DiLisio J., Burnett E. (2021). Adaptive selection of a prion strain conformer corresponding to established North American CWD during propagation of novel emergent Norwegian strains in mice expressing elk or deer prion protein. PLoS Pathog..

[130] Otero A., Duque Velasquez C., Johnson C., Herbst A., Bolea R., Badiola J.J., Aiken J., McKenzie D. (2019). Prion protein polymorphisms associated with reduced CWD susceptibility limit peripheral PrP(CWD) deposition in orally infected white-tailed deer. BMC Vet. Res..

[131] Nichols T.A., Nicholson E.M., Liu Y., Tao W., Spraker T.R., Lavelle M., Fischer J., Kong Q., VerCauteren K.C. (2021). Detection of two dissimilar chronic wasting disease isolates in two captive Rocky Mountain elk (*Cervus canadensis*) herds. Prion.

[132] Moore J., Tatum T., Hwang S., Vrentas C., West Greenlee M.H., Kong Q., Nicholson E., Greenlee J. (2020). Novel strain of the chronic wasting disease agent isolated from experimentally inoculated elk with LL132 prion protein. Sci. Rep..

[133] Holec S.A.M., Yuan Q., Bartz J.C. (2019). Alteration of prion strain emergence by nonhost factors. mSphere.

[134] Yuan Q., Eckland T., Telling G., Bartz J., Bartelt-Hunt S. (2015). Mitigation of prion infectivity and conversion capacity by a simulated natural process—repeated cycles of drying and wetting. PLoS Pathog..

[135] Kincaid A.E., Bartz J.C. (2007). The nasal cavity is a route for prion infection in hamsters. J. Virol..

[136] Jacquemot C., Cuche C., Dormont D., Lazarini F. (2005). High incidence of scrapie induced by repeated injections of subinfectious prion doses. J. Virol..

[137] Espinosa J.C., Andreoletti O., Castilla J., Herva M.E., Morales M., Alamillo E., San-Segundo F.D., Lacroux C., Lugan S., Salguero F.J. (2007). Sheep-passaged bovine spongiform encephalopathy agent exhibits altered pathobiological properties in bovine-PrP transgenic mice. J. Virol..

[138] Raymond G.J., Bossers A., Raymond L.D., O’Rourke K.I., McHolland L.E., Bryant P.K., Miller M.W., Williams E.S., Smits M., Caughey B. (2000). Evidence of a molecular barrier limiting susceptibility of humans, cattle and sheep to chronic wasting disease. EMBO J..

[139] Kocisko D.A., Priola S.A., Raymond G.J., Chesebro B., Lansbury P.T., Caughey B. (1995). Species specificity in the cell-free conversion of prion protein to protease-resistant forms: A model for the scrapie species barrier. Proc. Natl. Acad. Sci. USA.

[140] Gonzalez-Montalban N., Makarava N., Ostapchenko V.G., Savtchenk R., Alexeeva I., Rohwer R.G., Baskakov I.V. (2011). Highly efficient protein misfolding cyclic amplification. PLoS Pathog..

[141] Torres J.M., Espinosa J.C., Aguilar-Calvo P., Herva M.E., Relano-Gines A., Villa-Diaz A., Morales M., Parra B., Alamillo E., Brun A. (2014). Elements modulating the prion species barrier and its passage consequences. PLoS ONE.

[142] Harrathi C., Fernandez-Borges N., Erana H., Elezgarai S.R., Venegas V., Charco J.M., Castilla J. (2019). Insights into the bidirectional properties of the sheep-deer prion transmission barrier. Mol. Neurobiol..

[143] Priem J., Langeveld J.P., van Keulen L.J., van Zijderveld F.G., Andreoletti O., Bossers A. (2014). Enhanced virulence of sheep-passaged bovine spongiform encephalopathy agent is revealed by decreased polymorphism barriers in prion protein conversion studies. J. Virol..

[144] Wang Z., Qin K., Camacho M.V., Cali I., Yuan J., Shen P., Greenlee J., Kong Q., Mastrianni J.A., Zou W.Q. (2021). Generation of human chronic wasting disease in transgenic mice. Acta Neuropathol. Commun..

[145] Chianini F., Fernandez-Borges N., Vidal E., Gibbard L., Pintado B., de Castro J., Priola S.A., Hamilton S., Eaton S.L., Finlayson J. (2012). Rabbits are not resistant to prion infection. Proc. Natl. Acad. Sci. USA.

